# Characteristics of the home food environment that mediate immediate and sustained increases in child fruit and vegetable consumption: mediation analysis from the *Healthy Habits* cluster randomised controlled trial

**DOI:** 10.1186/s12966-015-0281-6

**Published:** 2015-09-17

**Authors:** Rebecca Wyse, Luke Wolfenden, Alessandra Bisquera

**Affiliations:** University of Newcastle, Callaghan, NSW Australia; Hunter New England Population Health, Hunter New England Local Health District, Wallsend, NSW 2287 Australia; Hunter Medical Research Institute, Newcastle, NSW Australia

**Keywords:** Mediation analysis, Home food environment, Fruit, Vegetable, Preschool children

## Abstract

**Background:**

The home food environment can influence the development of dietary behaviours in children, and interventions that modify characteristics of the home food environment have been shown to increase children’s fruit and vegetable consumption. However to date, interventions to increase children’s fruit and vegetable consumption have generally produced only modest effects. Mediation analysis can help in the design of more efficient and effective interventions by identifying the mechanisms through which interventions have an effect. This study aimed to identify characteristics of the home food environment that mediated immediate and sustained increases in children’s fruit and vegetable consumption following the 4-week *Healthy Habits* telephone-based parent intervention.

**Method:**

Analysis was conducted using 2-month (immediate) and 12-month (sustained) follow-up data from a cluster randomised control trial of a home food environment intervention to increase the fruit and vegetable consumption of preschool children. Using recursive path analysis, a series of mediation models were created to investigate the direct and indirect effects of immediate and sustained changes to characteristics of the home food environment (fruit and vegetable availability, accessibility, parent intake, parent providing behaviour, role-modelling, mealtime eating practices, child feeding strategies, and pressure to eat), on the change in children’s fruit and vegetable consumption.

**Results:**

Of the 394 participants in the randomised trial, 357 and 329 completed the 2- and 12-month follow-up respectively. The final mediation model suggests that the effect of the intervention on the children’s fruit and vegetable consumption was mediated by parent fruit and vegetable intake and parent provision of these foods at both 2- and 12-month follow-up.

**Conclusion:**

Analysis of data from the Healthy Habits trial suggests that two environmental variables (parental intake and parent providing) mediate the immediate and sustained effect of the intervention, and it is recommended these variables be targeted in subsequent home food environment interventions to bring about immediate and sustained changes in child fruit and vegetable intake.

**Trial registration:**

ACTRN12609000820202.

## Background

Consumption of adequate fruit and vegetables is important for good health and for the prevention of chronic disease [1, 2]. Despite this, the prevalence of inadequate fruit and vegetable intake among children is high [3, 4]. The home food environment has an important influence on the development of dietary behaviours in children [5]. A large body of cross-sectional research demonstrates associations between characteristics of the home food environment and children’s fruit and vegetable consumption. Systematic reviews synthesising these findings have concluded that parental fruit and vegetable consumption, fruit and vegetable availability and accessibility within the home, and mealtime practices such as not eating dinner in front of the television are positively associated with children’s fruit and vegetable consumption [6–8]. Given the importance of increasing children’s fruit and vegetable intake, interventions targeting the home food environment may hold promise [9, 10].

To date, the impact of interventions targeting children’s fruit and vegetable consumption have been modest [11–13]. Understanding the causal mechanisms by which interventions increase children’s fruit and vegetable consumption will enable the development of more effective and efficient interventions [14]. Mediation analysis is a useful analytical tool to identify causal relationships between variables and has been identified as fundamental to advance our understanding of how interventions work [14].

Despite recommendations for its routine use in the design, development, and evaluation of behavioral interventions [15] mediation analysis is rarely conducted as part of public health nutrition interventions. Previous mediation studies of childhood nutrition interventions have largely focused on psychosocial variables such as self-efficacy, attitudes and knowledge in school-aged children [16, 17]. However a recent study has investigated the role of maternal knowledge, diet, self-efficacy and feeding practices in mediating the diet quality of children aged under 2 years [18]. Across two systematic reviews of mediators of dietary change and obesity prevention interventions in school-aged children [16, 17], only three studies investigated potential mediators within the home food environment; two studies with primary school children [19, 20] and study one with adolescents [21]. None of these three studies investigated mediators of longer-term change, or investigated mediators at multiple time points, and as such, provide little evidence regarding the mechanisms by which sustained improvements in public health nutrition may be achieved through home-based interventions. Two of the three studies found partial evidence that parental consumption of fruit and vegetables mediated child consumption [19, 20]. The systematic reviews found insufficient evidence to support role modeling, eating together or availability as mediators of children’s dietary change [16, 17]. However, this is not surprising given the limited number of studies, and the caution of the review authors that the null findings may be attributable to limited statistical power, insufficiently sensitive measures, or a mediator with limited variability [16, 17].

Despite a previous study investigating mediators of consumption of non-core foods in 3–5 year olds [22], to the authors’ knowledge, no studies investigating the mediators of fruit and vegetable consumption in children of preschool-age (approximately 3–5 years) have been published. This represents an important area of research as children of this age; are at a different stage of development to children of school-age [23]; spend more time in the home environment; and are more reliant on their parents and carers for the provision of food [24]. These early years of childhood also represent a critical time in the development of dietary habits [25] that persist into adulthood [26]. Therefore, the aim of this study was to identify the characteristics of the home food environment that mediated increases in children’s fruit and vegetable consumption following an effective telephone-based parent intervention (*Healthy Habits*) [9, 10].

## Method

### Intervention overview

This mediation analysis was conducted on the 2- and 12- month follow-up results of a cluster randomised control trial of an intervention to increase fruit and vegetable consumption in 3 to 5 year-old children. The *Healthy Habits* intervention aimed to increase children’s fruit and vegetable intake by supporting parents to make changes to the home food environment [27], and increased children’s consumption at 2 months [9] and 12 months [10]. Ethical approval for the conduct of the trial was given by the Hunter New England Human Research Ethics Committee (08/10/15/5.09) and the University of Newcastle (H-2008-0410). The trial protocol [27] and short- [9] and long-term primary outcomes [10] are described in detail elsewhere, but briefly, participants were 394 parents of 3 to 5 year old children recruited from preschools within the Hunter region of New South Wales, Australia. Parents were randomly allocated to an intervention or control condition based on the preschool their child attended.

### Treatment conditions

#### Intervention

Parents allocated to the intervention condition received four 30-min telephone calls over a 4-week period. During the calls, a trained interviewer delivered a prewritten script using a Computer Assisted Telephone Interview (CATI) system. The script targeted three key areas of the home food environment: parental role-modeling of fruit and vegetable consumption; the availability and accessibility of foods in the home; and introducing supportive food routines, such as eating dinner as a family and without the television on. The intervention script incorporated a number of behaviour change techniques as classified in the taxonomy by Abraham and Michie [28] including goal-setting and review, self-monitoring of behaviour, barrier identification, identification as a role-model, and teaching to use prompts or cues. For example, parents were invited to set goals each week and participate in homework activities encouraging them to apply, directly into their home environment, the strategies and information covered in the telephone calls. Homework activities were tailored to the needs of participants, and were based on recommended home food environment practices. If the participant agreed to try the homework activity (e.g. storing pre-washed and chopped vegetables in the fridge), the interviewer guided them through the process of setting goals to help achieve this. Participants were instructed to record their homework tasks and goals for the week in a guidebook, which was posted out to them alongside a cookbook and a pad of meal planners.

#### Control

Parents allocated to the control group were mailed a printed booklet containing dietary advice for adults and children [29] and had no further contact until follow-up data collection.

#### Data collection

Data was collected from parents at baseline, and then 2, 6, 12 and 18 months later via telephone survey, administered by interviewers who were blind to group allocation. The first point of follow-up was deliberately scheduled for 2-months to allow for variation in time people took to complete the four call intervention. This paper reports the data from the 2- and 12- month follow-ups. Measures used to collect information about the primary trial outcome and the changes within the home food environment are described below.

### Measures

#### Primary outcome measure

Children’s fruit and vegetable consumption, was assessed using the fruit and vegetable subscale of the Children’s Dietary Questionnaire [30]. This scale provides a continuous outcome scored from 0 to 28 based on the variety and frequency of fruit and vegetable consumed over the past 24 h and past 7 days. The subscale has established reliability (Test-retest ICC = 0.75) and validity (Spearman correlation co-efficient = 0.58) on a comparable Australian sample, and has been recommended for use in intervention research [30].

#### Proposed mediating variables: characteristics of the home food environment

- Fruit and vegetable availability within the home (Availability): As no brief measure of home fruit and vegetable availability that was appropriate for telephone data collection could be sourced, to assess this, parents were read a list of 19 commonly consumed fruits and 24 commonly consumed vegetables from the Children’s Dietary Questionnaire [30]. The number of varieties of fruits and vegetables in their home at the time of the interview were then summed to provide a quantitative variable.
- Parental fruit and vegetable consumption (Parent Intake): Two items from the 1995 National Nutrition Survey [31] were included to assess the average number of serves of fruit and vegetables that parents consumed each day. Answers to these questions have been associated with biomarkers of fruit and vegetable consumption in a large Australian sample [32]. These items were summed to provide a single quantitative variable indicating the average combined serves of fruit and vegetables per day.
- Parental role-modeling of fruit and vegetable consumption (Role-modelling): Parents were also asked two items assessing the number of occasions (breakfast, morning tea, lunch, afternoon tea, dinner, after dinner) on the previous day that they had consumed fruit and vegetables in front of their child. These items were developed specifically for the study and were summed to provide a single quantitative variable that ranged from 0 to 12 role-modelling occasions per day.
- Providing behaviour of parents (Parent providing): Two additional questions were developed for the study to assess the number of occasions (breakfast, morning tea, lunch, afternoon tea, dinner, after dinner) on the previous day that the parent provided the child with fruit and vegetables, regardless of whether or not the child actually consumed these foods. These items were summed to provide a single quantitative variable that ranged from 0 to 12 providing occasions per day.
- Mealtime eating practices: Two items with established reliability were taken from the Healthy Home Survey to assess mealtime practices. *Eats together*–Parents were asked to indicate the number of days per week the family sits at a table to eat dinner together (range 0–7 days per week; Kappa = 0.73) [33]. *Television dinner*–Parents were also asked to indicate the number of days per week the child eats dinner in front of the television (range 0–7 days per week; Kappa = 0.80) [33].
- Child Feeding Strategies: Three items from the Healthy Home Survey assessed parents’ use of eating policies that encouraged fruit and vegetable consumption. On a five-point likert scale (‘all of the time’ to ‘never’) parents were asked how frequently they did the following: reward their child with desserts, snacks or confectionary if they finish their dinner; ask their child to eat everything on their plate at dinner; and allow their child to eat only at set meal times (intraclass correlation coefficient = 0.75, 0.79, 0.52) [33]. Items were recoded and scored 1 if the practice was thought to facilitate child fruit and vegetable consumption, and 0 if otherwise. Given evidence that some feeding strategies that encourage children to consume specific foods can increase the child’s dislike for those foods [34], the strategies of using dessert as a reward for finishing dinner and asking children to eat everything on their plate were recoded so that responses of “never” or “rarely” was scored 1. Given evidence suggests generally allowing children to eat only at set meal and snack times is associated with increased consumption of fruit and vegetables [35], responses of “all of the time” and “most of the time” were recoded 1.
- Pressure to eat (Pressure): The ‘Pressure to Eat’ subscale from Birch’s Child Feeding Questionnaire, demonstrated to be internally consistent and reliable, was included to measure the extent to which parents try to control the amount and type of food eaten by their child, where a higher score indicates more pressure [36, 37]. The score of the four items that comprise this scale were averaged.
- Fruit and vegetable accessibility within the home (Accessibility): To assess vegetable accessibility, parents were asked whether vegetables were stored in a form that facilitated their consumption, for example, washed and chopped (Item reliability kappa = 0.57, Item validity kappa = 0.43) [33]. To assess fruit accessibility, the vegetable question was adapted, ‘Do you have any ready to eat fresh fruit on a shelf in the refrigerator or on the kitchen counter now, for example, fruit you have washed or chopped to make ready to eat, like bunches of grapes, berries, or oranges’? These items were scored 1 (=yes) and 0 (=no) and then summed to provide a single score (range 0–2).

### Analysis

**Fig. 1:**
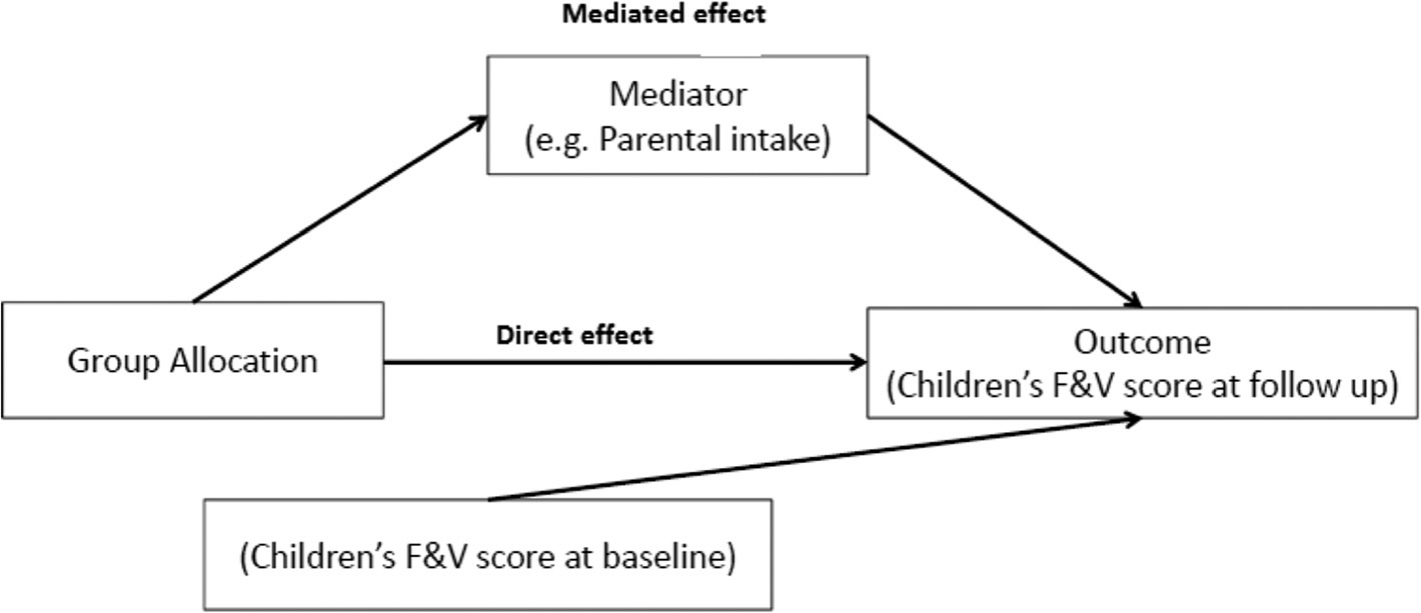
Example of path analysis model with single mediator (at a single time point)

**Fig. 2:**
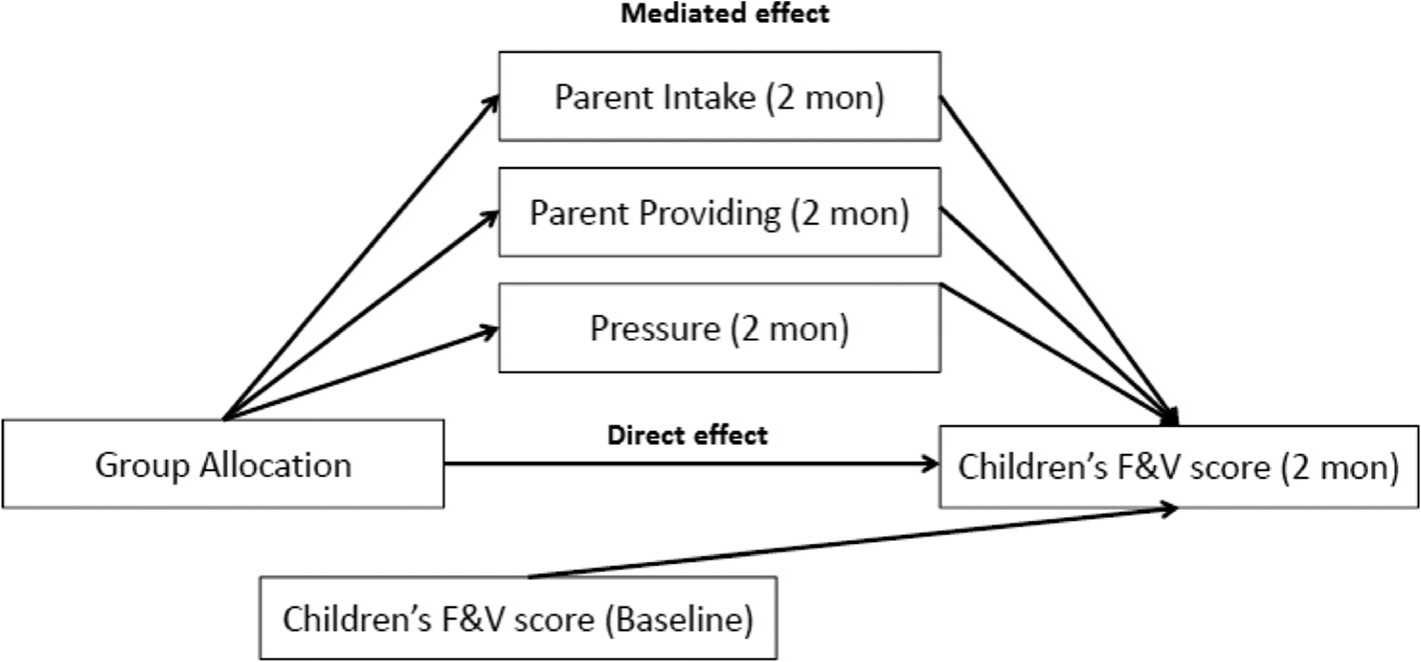
Combined model: Path analysis model with all mediators included (at a single time point)

**Fig. 3:**
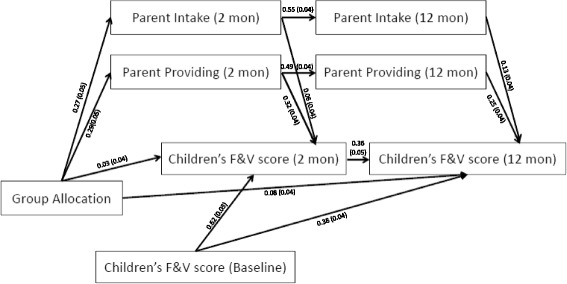
Final mediation model

Data were analysed using SAS version 9.2 (SAS Institute, Cary, North Carolina, USA) and only participants with complete data for all variables used at each timepoint were included in the analysis. Continuous variables with skewed distribution were dichotomised based on previously used cut-points [35]; and change scores were calculated for all other continuous variables (i.e. 2-month score–baseline score; 12-month score–baseline score). Firstly, associations between each of the proposed mediators and group allocation (intervention or control) were tested at each time point, with *p*-values calculated using generalised estimating equations to account for clustering of children within preschools. However, as the intraclass correlation coefficients were too small to have any impact on the findings (0.003 at 2 months and negative at 12 months), further consideration of correlation between data points was dropped from subsequent analyses. Where a variable showed preliminary evidence of an association with group allocation (*p* < 0.05), the direct and indirect effects of the intervention on children’s fruit and vegetable score were examined using recursive path analysis where the paths were estimated using multiple linear regression. Standardised coefficients were calculated for all estimates so that all direct and indirect (mediated) effects could be interpreted and compared on the same scale. Mediators were tested one at a time and if there was evidence of an indirect effect of group allocation on the outcome through the mediator (Fig. 1) at either time point, then the mediator was entered into a combined model that incorporated all significant mediators within a single time point (Fig. 2). Variables that were not significant in the combined models were removed to create the final mediation model (Fig. 3). The final mediation model incorporated variables from both follow-up time points, as well as the 2-month outcome (child fruit and vegetable consumption at 2-months). This was to acknowledge that some of the change at 12-months would be the result of the immediate intervention effect at 2-months. Incorporating variables from both time points allowed the additional intervention effect at 12-months to be isolated from the intervention effect that was sustained from the 2-month follow up. To assess how well the data fit the models, the following statistics were calculated (with the threshold for a ‘good’ fit reported in brackets); Adjusted Goodness Of Fit (AGF >0.95), Root Mean Square Error of Approximation (RMSEA <0.05), and Standardised Root Mean Square Residual (SRMSR <0.05). Due to the differences in the units across variables, standardised effects and standard errors are given for ease of comparison.

## Results

Of the 394 participants (208 intervention, 186 control) that completed the baseline assessment, 357 completed the 2-month follow up (178 intervention, 179 control), and 329 completed the 12-month follow up (165 intervention, 164 control). Baseline characteristics of the intervention and control group have been reported elsewhere [9], but briefly, the parent sample was as follows: 96 % female, 47 % university educated, 41 % with a household income greater than AU$ 100,000; and an average age of 35.4 years (SD = 5.4); with an average 2.3 children under 16 years (SD = 0.8). The children who were the target of the intervention had an average age of 4.3 years (SD = 0.6) and 49 % were female. There were no significant differences at baseline between the intervention and control groups on the above characteristics. Three hundred fifty six provided a complete set of data at 2-months, and 327 provided a complete set of data at 12-months.

At both time points, there was a significant difference in children’s fruit and vegetable consumption between intervention and control groups [9, 10].

Table 1 shows that the intervention group has higher parent fruit and vegetable intake at 2 months compared to the control group. At 12-months, the frequency of providing children with fruit and vegetables was higher and the pressure to eat score was lower among intervention parents. These variables were entered into a series of single mediation models (Fig. 1), the path analyses for which is reported in Tables 2 and 3.

**Table 1:** Differences in proposed mediators between treatment groups at 2-month and 12-month follow-up

			2-months			12-months		
			Treatment		*p*-value	Treatment		*p*-value
	Proposed mediator	Category	Intervention (*n* = 208)	Control (*n* = 186)		Intervention (*n* = 208)	Control (*n* = 186)	
Change score	Availability	mean (SD)	1.0 (4.7)	0.7 (4.4)	0.4721	1.1 (4.2)	0.3 (5.2)	0.3437
	Parent Intake (F&V serves/day)	mean (SD)	1.0 (1.8)	0.1 (1.5)	<0.0001	0.6 (2.0)	0.0 (1.7)	0.1459
	Role modelling (Times/day)	mean (SD)	0.5 (1.7)	0.4 (1.5)	0.3312	−0.0 (1.9)	0.0 (1.4)	0.7426
	Parent provision (Times/day children provided F&V)	mean (SD)	1.1 (1.7)	0.4 (1.4)	0.0006	0.7 (1.5)	0.3 (1.4)	0.0232
	Pressure	mean (SD)	−0.2 (0.6)	−0.1 (0.6)	0.1339	−0.2 (0.6)	0.0 (0.7)	0.0413
Single point in time	Eats together (as a family at a table/bench)	Everyday (=1) 0–6 days/week (=0)	108 (60 %)	109 (61 %)	0.7023	104 (63 %)	99 (61 %)	0.2920
	Television dinner (Eat dinner in front of the TV)	1–7 days/week (=1) Never (=0)	91 (51 %)	89 (49 %)	0.5435	77 (47 %)	77 (48 %)	0.4788
	Accessibility							
	- Neither F nor V	0.0	24 (14 %)	33 (18 %)	0.2039	27 (16 %)	26 (16 %)	0.4761
	- F or V, not both	1.0	64 (36 %)	85 (47 %)		57 (35 %)	66 (41 %)	
	- Both F & V	2.0	88 (50 %)	61 (34 %)		80 (49 %)	68 (43 %)	
	Reward child with desserts etc. if finish plate at dinner?	Never/rarely	82 (46 %)	60 (33 %)	0.0557	77 (47 %)	64 (40 %)	0.6543
	Ask child to eat everything on plate at dinner	Never/rarely	70 (39 %)	42 (23 %)	0.1481	48 (29 %)	45 (28 %)	0.6901
	Generally allow child to eat only at set meal times	All/most of the time	75 (42 %)	74 (41 %)	0.7101	72 (44 %)	65 (40 %)	0.2796

**Table 2:** Direct and mediated effects at 2 months

		Standardised effects of treatment on outcome (se)			
Outcome	Mediator	Direct effect	*p*-value	Mediated effect	*p*-value
Children’s F&V score (2 months)	Parent intake (2 months)^a^	0.12 (0.04)	0.0049	0.02 (0.01)	0.0564
	Parent provision (2 months)^a^	0.06 (0.04)	0.1010	0.07 (0.02)	<0.0001
	Pressure (2 months)^a^	0.14 (0.04)	0.0009	0.00 (0.00)	0.5512
	Combined model (2 months)^b^	0.05 (0.04)	0.2512	0.09 (0.02)	<0.0001

Fit statistics: AGFI = 0.9745, RMSEA = 0.0290, SRMSR = 0.0336

^a^See Fig. 1 for example

^b^See Fig. 2 for example

**Table 3:** Direct and mediated effects at 12 months

		Standardised effects of treatment on outcome (se)			
Outcome	Mediator	Direct effect	*p*-value	Mediated effect	*p*-value
Children’s F&V score (12 months)	Parent intake (12 months)^a^	0.16 (0.04)	0.0002	0.02 (0.01)	0.0315
	Parent provision (12 months)^a^	0.14 (0.04)	0.0006	0.04 (0.02)	0.0169
	Pressure (12 months)^a^	0.17 (0.04)	0.0002	0.01 (0.01)	0.2467
	Combined model (12 months)^b^	0.11 (0.04)	0.0067	0.06 (0.02)	0.0025

Fit statistics: AGFI = 0.9432, RMSEA = 0.0726, SRMSR = 0.0574

^a^See Fig. 1 for example

^b^See Fig. 2 for example

### Mediation effects at 2 months

Table 2 shows that the effect of treatment allocation on children’s F&V score at 2 months is mediated by parental provision (*p*-value <0.0001), with the mediated effect (estimate = 0.07; s.e = 0.02) being larger than the direct effect (estimate = 0.06; s.e = 0.04) of the intervention. The combined model (which includes all three mediators; see Fig. 2) also shows that most of the effect of the treatment on children’s F&V score at 2 months is mediated by parental intake, provision and pressure (estimate 0.09; s.e = 0.02; *p*-value <0.0001).

### Mediation effects at 12 months

Table 3 shows that effect of treatment allocation on children’s F&V score at 12 months is mediated by parental provision (*p*-value = 0.0169) and parental intake (*p*-value = 0.0315), however the mediated effect is small compared to the direct effect. The combined model shows that approximately one third (estimate = 0.06; s.e = 0.02) of the total effect of the intervention on the outcome is mediated by intake, provision and pressure, and the rest (estimate = 0.11; s.e = 0.04) is due to the direct effect.

### Mediation effects at 2 and 12 months

Given parent intake and parent provision of fruit and vegetables were the only mediators identified in the path analysis, redundant variables (i.e. parent pressure) were removed to create a final mediation model that incorporated child fruit and vegetable consumption at both 2-months and 12-months (Fig. 3). The analysis appears in Table 4.

**Table 4:** Final Mediation Model–direct and mediated effects of treatment (group allocation) and each mediator on 12-month outcome (child fruit and vegetable consumption score)

Standardised effects of treatment & mediators on 12-month outcome (child fruit and vegetable consumption score) (se)				
Variable	Direct effect	*p*-value	Mediated effect	*p*-value
Group allocation	0.08 (0.04)	0.0274	0.11 (0.02)^a^	<0.0001
Parent provision (2 months)	0		0.24 (0.03)^b^	<0.0001
Parent provision (12 months)	0.25 (0.04)	<0.0001	0	
Parent intake (2 months)	0		0.10 (0.03)^c^	0.0002
Parent intake (12 months)	0.13 (0.04)	0.0003	0	
Children’s F&V score (2 months)	0.36 (0.05)	<0.0001	0	

Fit statistics: AGFI = 0.9434, RMSEA = 0.0563, SRMSR = 0.0560

^a^this is the effect of group allocation on the 12 months F&V consumption that is mediated through parent provision and intake at 2 and 12 months, and F&V consumption at 2 months

^b^this is the effect of parent provision at 2 months on 12 months F&V consumption that is mediated through parent provision at 12 months and F&V consumption at 2 months

^c^this is the effect of parent intake at 2 months on 12 months F&V consumption that is mediated through parent intake at 12 months and F&V consumption at 2 months

The final model (Table 4) suggests that treatment (e.g. random allocation to the intervention group) has a direct effect on child fruit and vegetable consumption score at 12 months (*p* = 0.0274). The effect of the intervention on the outcome is also mediated by parent intake (*p* = 0.0002) and parent provision (*p* < 0.0001), indicating that treatment influences the change in parent provision and parent intake at 2 months, which in turn influences the same variables and the outcome at 12 months. The greatest predictor of sustained children’s fruit and vegetable consumption was the ‘immediate’ change in consumption, with the 2-month outcome having the largest direct effect (0.36) of any included variable. In terms of the relative contributions of the 12-month mediators, parental provision was a stronger mediator than parental intake, with the standardised indirect effect of the former variable (0.25) about double that of the latter (0.13). Goodness of Fit indices suggest that this model provided an adequate fit to the data.

## Discussion

Mediation analysis of data from the *Healthy Habits* trial of a parent intervention to increase preschooler fruit and vegetable intake suggests that two home food environment variables (parent fruit and vegetable intake and parent provision of fruit and vegetables) mediate the immediate intervention effect (at 2 months) as well as the sustained intervention effect (at 12 months). The greatest predictor of an increase in children’s fruit and vegetable consumption at 12-months was children’s consumption at 2-months.

Given the relative paucity of mediation studies of children’s dietary change, especially among preschool-aged children, this study makes an important contribution to the evidence base.

The findings of this study suggest that interventions to increase preschool children’s fruit and vegetable consumption both in the short- and long-term, should focus on increasing levels of parent fruit and vegetable intake as well as increasing the frequency with which parents provide fruit and vegetables to their children throughout the day. The importance of parental fruit and vegetable intake is consistent with previous association studies as well as theoretical models including the approach advocated by Golan and Weizman [38]. Interventions that incorporate strategies to more directly increase parent fruit and vegetable intake, such as through the use of parent self-monitoring of consumption and increasing knowledge of adult intake recommendations, may further enhance the long-term impact of child-based dietary interventions.

These significant mediators of intervention effect on child fruit and vegetable consumption in this study are in contrast to a prior mediation analysis from the same trial examining characteristics of the food home environment in relation to children’s consumption of non-core foods. Specifically, in the current study, the impact of the intervention on short-term (2-month) children’s fruit and vegetable consumption was mediated by parental consumption of fruit and vegetables and parent provision of fruit and vegetables, where children’s consumption of non-core foods was mediated by child feeding strategies (such as restricting or rewarding with dessert, and finishing dinner or seconds policies) and accessibility to non-core foods [22]. Such findings suggest that the mechanism by which interventions may influence child diet may differ depending on the aspect of child diet that is being targeted. Furthermore, the findings suggest that efforts to maximise the efficacy of fruit and vegetable intentions by removing intervention strategies that do not mediate intervention effects could have unintended adverse effects on other aspects of child diet. Given systematic review findings have highlighted that the use of measures with unacceptable or unknown psychometric properties is a limitation within the literature [16], additional studies using robust measures of hypothesised mediators, measured at multiple time points, and analysis of multiple dietary outcomes simultaneous would be valuable contribution for future mediation research.

The findings from the current study should be considered in relation to the study limitations. Characteristics of the home food environment have been inconsistently defined, and measured across the literature [5]. While attempts were made to use valid and reliable measures, these were not always available or feasible to administer over the telephone. As such some items were either adapted from existing items or developed specifically for this trial. Furthermore, dichotomising skewed continuous variables may have affected the ability to detect mediation effects. It is also acknowledged that there could be other mediators (environmental or otherwise) of children’s fruit and vegetable consumption that were not considered in these models and that may influence the change in children’s fruit and vegetable consumption. Nonetheless, the randomised trial design is a key strength of the study. The design enables us to assume no associations exist between the treatment (allocation group) and children’s fruit and vegetable consumption at baseline. Other strengths include the relatively large sample, extended follow-up, and the inclusion of baseline fruit and vegetable consumption in the models. A further strength is the inclusion of mediators at multiple points in time within the final model, which allows immediate mediation effects that are sustained over time to be distinguished from mediator effects that commence later, and represents a novel contribution to the literature.

## Conclusion

This investigation of characteristics of the home food environment that mediated increases in preschool children’s fruit and vegetable consumption, following parent participation in a randomised controlled trial, identified two mediating variables: parental fruit and vegetable intake, and the frequency with which parents provide fruit and vegetable to their children throughout the day. It is recommended future home-based interventions to increase children’s fruit and vegetable consumption target these variables. Mediation analysis has great potential to advance the field of dietary intervention and it is advised that mediation analysis be included as a standard part of intervention efficacy trials. Additional research into potential mediators within the home food environment would guide the development of future dietary interventions for young children and advance the science of public health nutrition.
